# Implementing new health interventions in developing countries: why do we lose a decade or more?

**DOI:** 10.1186/1471-2458-12-683

**Published:** 2012-08-21

**Authors:** Alan Brooks, Thomas A Smith, Don de Savigny, Christian Lengeler

**Affiliations:** 1 Swiss Tropical and Public Health Institute, Socinstrasse 57, Basel, CH-4002, Switzerland; 2 University of Basel, Petersplatz 1, Basel, CH-4003, Switzerland

**Keywords:** Vaccine, Malaria, Intervention, Research and development, Implement, Access, Developing country

## Abstract

**Background:**

It is unclear how long it takes for health interventions to transition from research and development (R&D) to being used against diseases prevalent in resource-poor countries. We undertook an analysis of the time required to begin implementation of four vaccines and three malaria interventions. We evaluated five milestones for each intervention, and assessed if the milestones were associated with beginning implementation.

**Methods:**

The authors screened World Health Organization (WHO) databases to determine the number of years between first regulatory approval of interventions, and countries beginning implementation. Descriptive analyses of temporal patterns and statistical analyses using logistic regression and Cox proportional hazard models were used to evaluate associations between five milestones and the beginning of implementation for each intervention. The milestones were: (A) presence of a coordinating group focused on the intervention; (B) availability of an intervention tailored to developing country health systems; (C) international financing commitment, and; (D) initial and (E) comprehensive WHO recommendations. Countries were categorized by World Bank income criteria.

**Results:**

Five years after regulatory approval, no low-income countries (LICs) had begun implementing any of the vaccines, increasing to an average of only 4% of LICs after 10 years. Each malaria intervention was used by an average of 7% of LICs after five years and 37% after 10 years. Four of the interventions had similar implementation rates to hepatitis B vaccine (HepB), while one was slower and one was faster than HepB. A financing commitment and initial WHO recommendation appeared to be temporally associated with the beginning of implementation. The initial recommendation from WHO was the only milestone associated in all statistical analyses with countries beginning implementation (relative rate = 1.97, P < 0.001).

**Conclusions:**

Although possible that four milestones were not associated with countries beginning implementation, we propose an alternative interpretation; that the milestones were not realized early enough in each intervention’s development to shorten the time to beginning implementation. We discuss a framework built upon existing literature for consideration during the development of future interventions. Identifying critical milestones and their timing relative to R&D, promises to help new interventions realize their intended public health impact more rapidly.

## Background

The GAVI Alliance (GAVI; formerly the Global Alliance for Vaccines and Immunization) and the Global Fund to Fight AIDS, Tuberculosis and Malaria (GFATM) were established in 2000 and 2002, respectively. Since then, they have committed more than USD 20 billion to address the divergence in health status and access to health interventions between developed and developing countries (DCs). In parallel, the past decade has seen unprecedented investments in research and development (R&D) for new health interventions for use in developing countries; approximately USD 3.2 billion was invested in 2009 alone, an increase of 8.2% from 2008 [1]. Product development partnerships (PDPs) have grown in number and are focused on developing drugs, rapid diagnostic tests, vaccines and other interventions for developing countries [2]. The use of such interventions might eventually be subsidized by GAVI, GFATM, and other financing mechanisms.

It is normal in R&D for a large percentage of candidate interventions to fail during development. However, the investments above will lead to some new interventions being approved by regulators and becoming available for use. A critical step is then how quickly they will begin to be implemented through national health systems of developing countries. Beginning implementation does not guarantee, but is on the critical path to, interventions becoming widely accessible to people in need in developing countries.

**Figure 1:**
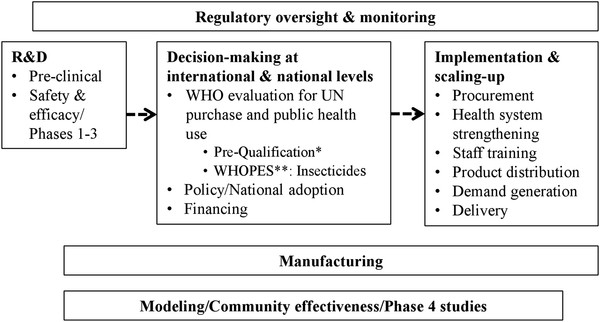
**New interventions: From R&D to implementation (illustrative).** *Drugs, Vaccines Diagnostics, Reproductive health supplies. **WHOPES: WHO Pesticide Evaluation Scheme

The progression from R&D to implementation through national health systems in developing countries can be broken down into a series of stages and parallel activities, although it may vary somewhat by type of intervention (Figure 1). An intervention moves from R&D through a series of international and ultimately national policy decisions about use and financing. These decisions lead to the beginning of implementation, scaling-up over months or years to target levels, and ideally access as defined below. Regulatory, manufacturing, and supplemental study activities take place in parallel. The World Health Organization (WHO) plays a critical role, such as reviewing interventions to determine if they are suitable for purchase by agencies of the United Nations (UN) [3,4]. Figure 1 presents a process following a linear progression for clarity, however this may rarely be the actual case. The process varies according to the unique scientific, policy and other challenges of each intervention. 

Access has been defined as the result of a set of coordinated activities needed to ensure that interventions will ultimately have an equitable public health impact [5]. A number of authors have studied access independently. Obrist *et al.* (2007) proposed an access framework focusing on consumer decisions, livelihood, and the assets of poor populations with regard to health interventions [6]. They reviewed five concepts determining access to health interventions: availability, accessibility, affordability, adequacy, and acceptability. Mahoney *et al.* (2007) identified four criteria for access to new vaccines: availability; affordability; acceptability; and adoptability [7]. They propose an access framework that acknowledges the role of decisions made during the R&D period on later implementation. PDPs generally agree on a similar set of access criteria relevant to many interventions [5,8].

**Figure 2:**
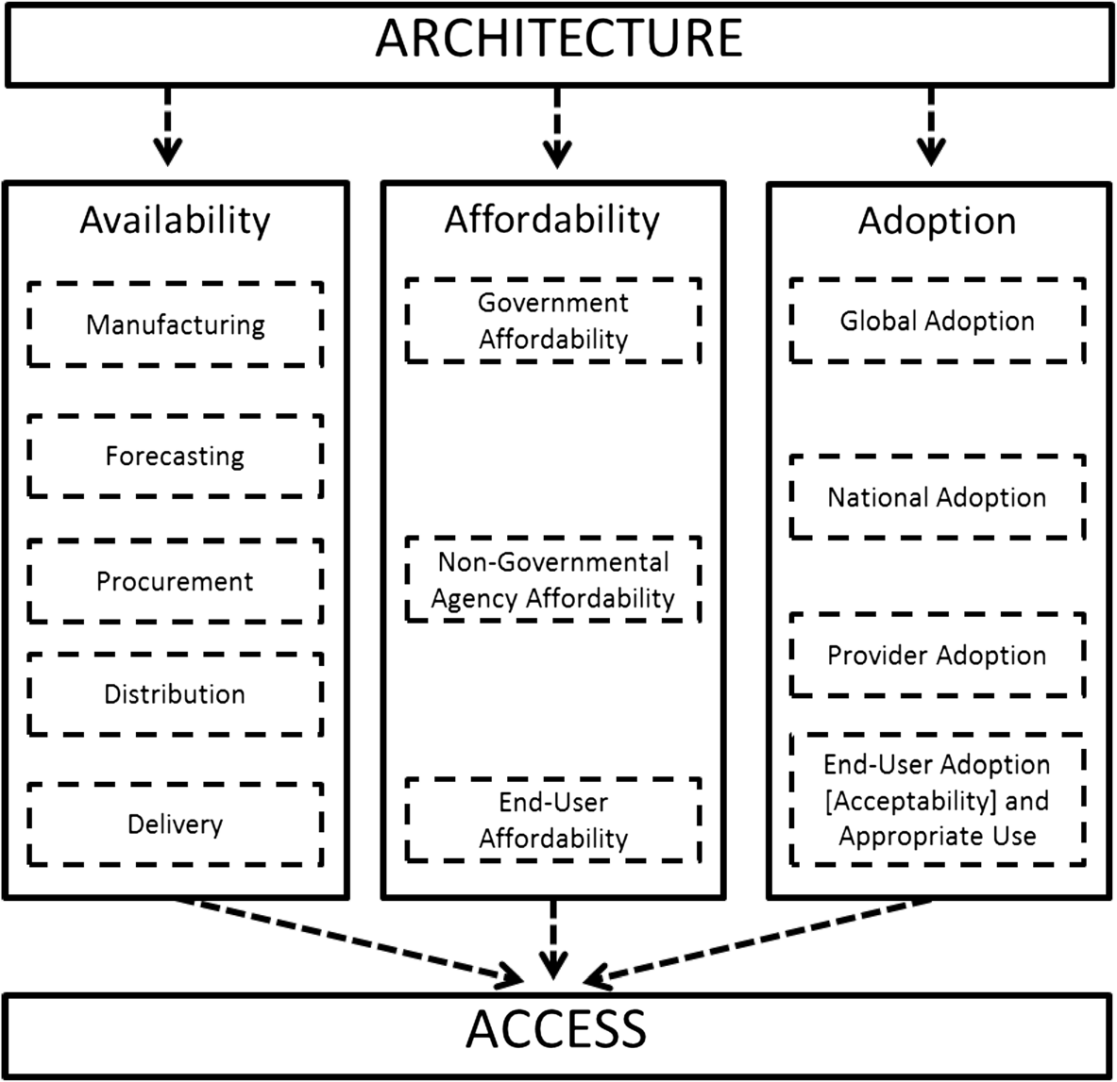
**Frost and Reich’s (2008) access framework.** The figure presents access as depending on a coordinating architecture that ensures that availability, affordability and adoption considerations are addressed for an intervention. Architecture: Organizational structures and relationship established with the purpose of coordinating and steering the availability, affordability, and adoption activities. Availability: Logistics of making, ordering, shipping, storing, distributing, and delivering a new health technology to ensure it reaches the hands (or mouths) of the end-user. Affordability: Ensuring that health technologies and related services are not too costly for the people who need them. Adoption: Gaining acceptance and creating demand for a new health technology from global organizations, government actors, providers and dispensers, and individual patients. The concept of “acceptability” is inherent in “End-User Adoption and Appropriate Use” but was made explicit in the graphic above to illustrate this framework’s consistency with the work of other authors. Reproduced under a Creative Commons Attribution-Noncommercial-Share Alike 3.0 License [8]

Frost & Reich (2008) analyzed the history of access to six health interventions in the developing world: praziquantel; hepatitis B vaccine; malaria rapid diagnostic tests; Norplant; vaccine vial monitors; and female condoms [8]. They proposed that access depends on activities related to four key factors: architecture, availability, affordability, and adoption. Architecture encompasses the organizational structures and relationships that coordinate activities addressing availability, affordability and adoption (Figure 2). They also provide a historical description of the R&D phase of each intervention, noting that developer choices are important for later policy decisions on use and implementation. Each concept Mahoney *et al.* (2007), Obrist *et al.* (2007), and PDPs use in their access frameworks is consistent with the ones identified by Frost & Reich [5-8]. For example, Obrist’s *et al.* concept of acceptability is consistent with Frost and Reich’s “end-user adoption and appropriate use”. The one exception, which is not relevant to national implementation of an intervention and therefore this analysis, is Obrist *et al.*’s adequacy concept, matching health service organization with individual client expectations [6]. 

These analyses of access are complemented by many analyses of strategies for the implementation of single or closely-related interventions [9,10]. Some of the literature focuses on the R&D period, including how clinical trials and regulatory processes impact implementation [11-15]. Much of the literature focuses on the period after regulatory approval, using qualitative [7,16-25] and quantitative approaches [26-30] (Table 1). 

**Table 1:** Considerations affecting access to new interventions

	**Relevance to access**	**Considerations**
**Prior to regulatory approval**	Availability & Affordability	Design of interventions specifically for the needs of DCs [31-37]
	Adoption	Clinical studies to address questions unique to DCs [12,38-42]
		Requirements for international policy recommendations [43,44]
		Preparing for country decision-making processes [45]
**After regulatory approval**	Coordinated action	Coordination between stakeholders [16]
	Availability	Alignment of intervention with the unique needs of developing country health systems [16,18,19,23,24]
		Forecasting and manufacturing plans incorporating DCs, [16,21]
		Adapted procurement mechanisms [16,21]
	Affordability	Affordability, financing, & cost-effectiveness [16,18,19,21-24,27-30]
	Adoption	Research aligned with policy-maker needs, including burden of disease addressed by an intervention [18,21-24,29]
		Importance of international technical consensus and recommendations, including influence of neighboring countries [21,23,46]
		Strengthened national processes, acceptability, and/or governance [17,19-21,26]

Legend: Pubmed and Web of Science® databases were searched for full names or abbreviations of hepatitis B, *Haemophilus influenzae* type B, pneumococcal conjugate, rotavirus, insecticide-treated net, rapid diagnostic test, or artemisinin-based combination therapies AND (malaria or vaccin*) AND (develop* OR decision* OR policy* OR adopt* OR implement*).

This paper hypothesizes that five high-level milestones, consistent with the research on access above, are associated with developing countries beginning implementation of interventions. If these milestones are associated, it follows that they could be targets of efforts by those developing new interventions in order for implementation to begin, and access to be achieved, more quickly. This paper explores this hypothesis by analyzing how long it took after regulatory approval, or equivalent, for countries to begin implementing each of seven interventions, and related implementation to when each of these five milestones took place for each intervention.

## Methods

The number of years between initial approval of seven interventions by a stringent regulatory body and the beginning of implementation, considered to be the first year of reported use by each country through its national health system, was estimated from available data. Similar but more limited analyses have been applied to health interventions previously [16,47,48]. In addition, the timing of each of five hypothesized milestones was determined for each intervention. Descriptive and statistical analyses were used to assess associations between the timing of milestones and countries beginning implementation.

The year of approval by a stringent regulatory authority was intended to reflect the earliest time point at which it would have been ethical to begin implementation on a large scale outside of a controlled trial. This is especially critical for vaccines and drugs because of the issues of safety and quality. Some interventions, such as ITNs, while generally not overseen by regulatory authorities, have mechanisms in place for reviewing safety and more recently quality, which were considered as equivalent to the time of regulatory approval for this analysis [49,50]. In these cases, efficacy and effectiveness may be evaluated through the establishment of a scientific consensus between experts on the basis of existing trial experience [51,52].

Countries were categorized as low- (LIC), lower-middle- (LMIC), upper-middle- (UMIC), and high-income (HIC) according to the World Bank stratification, corresponding respectively to 2009 gross national income per capita of $995 or less, $996 - $3,945, $3,946 - $12,195, and $12,196 or more [53]. World Bank data were used as they provide a standardized categorization for countries, and are used by the GFATM in determining support to countries.

### Estimating time from regulatory approval, or equivalent, to beginning implementation

#### Vaccines

Four vaccines were selected for inclusion in the study based on their public health importance, diversity in year of availability, similar ages of target populations and comparable delivery strategies. The diseases they target — hepatitis, pneumonia, meningitis, and diarrhea— are among the world’s leading causes of mortality and morbidity, especially in developing countries. Hepatitis B (HepB) and *Haemophilus influenzae* type b (Hib) vaccines have been available for decades while pneumococcal conjugate (PC) and rotavirus (RV) vaccines are among the newest.

The regulatory approval of the first rotavirus vaccine was a unique case. Licensed in 1998, it was removed from the market in 1999 due to concerns about intussusceptions (a potentially life threatening telescoping of the intestine within itself). A new rotavirus vaccine was licensed in 2004 in Mexico although WHO did not consider its NRA fully functional. The vaccine was then licensed by the European Medicines Agency (EMA) in 2006 [54]. This analysis considered 2006 to be the year of the rotavirus vaccine’s first stringent regulatory approval, although the history is considered further in the discussion.

WHO collects reports from 193 countries each year in order to assess vaccine implementation [55] (Table 2). These data were used to generate tables showing the first year of vaccine use and the number of years until coverage matched that of the third dose of diphtheria-tetanus-whole cell pertussis vaccines (DTP3), which is given to the same infant population at the same times as the other vaccines in this analysis. WHO’s data cover the years 1989–2009 for HepB, 1991–2009 for Hib, and 2008–09 for PC and RV. Data for the remaining years through March, 2011 and for missing dates in the WHO data were taken from the Vaccine Information Management System (VIMS), a database maintained at the International Vaccine Access Center, Johns Hopkins University [56]. 

**Table 2:** Characteristics of countries included in the analysis and summary of responses

	**Included in sample**	**High- income**	**Upper- middle- income**	**Lower- middle- income**	**Low- income**	**No income category**	**Intervention implemented (%)**	**Not implemented**	**Did not respond**
**Hepatitis B vaccine**	193	50	46	54	40	3	180 (93%)	13	0
***Haemophilus influenzae*type B vaccine**	193	50	46	54	40	3	163 (84%)	30	0
**Rotavirus vaccine**	193	50	46	54	40	3	30 (16%)	163	0
**Pneumococcal vaccine**	193	50	46	54	40	3	61 (32%)	132	0
**Insecticide-treated mosquito net**	104	4	21	39	40	0	89 (86%)	0	15
**Rapid diagnostic test**	104	4	21	39	40	0	40 (38%)	6	58
**Artemisinin-based combination therapy**	104	4	21	39	40	0	63 (61%)	12	29

#### Malaria interventions

Malaria was selected for inclusion as it is one of the major causes of mortality and morbidity in children, and preventive, therapeutic and diagnostic interventions are available. Insecticide-Treated Nets (ITNs) and more recently developed Long-Lasting Insecticidal Nets (LLINs) prevent malaria. In this paper, “ITN” is used for both ITNs and LLINs. Immuno-chromatographic rapid diagnostic tests (RDTs) allow diagnosis of malaria with minimal training and hence are valuable for optimizing treatment strategies. Artemisinin-based combination therapies (ACTs) are the current standard for malaria treatment.

In the absence of a formal regulatory structure, regulatory approval of ITNs was based on a WHO expert committee concluding they were safe for individuals, and therefore could be used outside clinical trials [49]. For RDTs, regulatory approval was considered to be the point at which the first RDT became available in the developed world where there are strong quality assurance systems.

WHO provided data on country implementation in 104 malaria-endemic countries, taken from the 2010 survey of countries by the Global Malaria Program as part of the annual World Malaria Report [57] (Table 2). The survey asked respondents to identify the year WHO-recommended malaria policies began to be implemented. Respondents indicated the first year “ITNs distributed to all age groups” or “ITNs distributed free of charge”. For diagnostic tests, respondents indicated the first year “RDTs used in communities”, and for artemisinin-based combination therapies they indicated the first year “ACT is free of charge for under 5 years olds in the public sector” or “ACT is free to all”. Non-response to the specific questions about implementing the interventions, while other questions in the survey were answered, was classified as not implementing the intervention. Fourteen percent of countries did not respond for the ITN questions, 56% for RDTs, and 28% for ACTs. The earliest date was used in cases where different dates were given for each policy or if parts of countries (e.g. mainland Tanzania versus Zanzibar) reported different dates.

### Five hypothesized milestones

Milestones consistent with and thought to be important to coordination, availability, affordability and adoption were hypothesized from the literature above (Table 1). This is not to suggest that these hypothesized milestones addressed all aspects of each access concept, nor that they always happened in a specific sequence. The timing and source document for each milestone is summarized in Table 3.

**Table 3:** Hypothesized access milestones for each intervention

	**Regulatory approval**	**A) Coordinating group**	**B) Improved intervention**	**C) Financing commitment**	**D) Initial WHO recommendation**	**E) Comprehensive WHO recommendation**
**HepB**	1982	1986	1996	2000	n/a	1992
**Hib**	1988	1998	1997	2000	1998	2006
**RV**	2006	2003	2008	2007	2007	2009
**PC**	2000	2003	2009	2007	n/a	2007
**ITN**	1991	1998	2001	2002	1995	2007
**RDT**	1995	2003	n/a	2002	2006	2010
**ACT**	1999	1999	2009	2002	2002	2006

**Legend**.

Data sources below relate to the column number for each intervention. Websites were accessed on April 14, 2011 unless otherwise indicated.

**Hepatitis B vaccine:** Regulatory approval) [58]; A) [59]; B) Personal communication, Marie-Claude Dubois, April 11, 2011; C) [60]; D) n/a; E) [61].

***Haemophilus influenzae*type b vaccine:** Regulatory approval) [43]; A) [62]; B) Personal communication, Marie-Claude Dubois, April 11, 2011; C) [63]; D-E) [43].

**Rotavirus vaccine :** Regulatory approval) [54]; A) [64]; B) [65]; C) [66]; D-E) [43].

**Pneumococcal conjugate vaccine**: Regulatory approval) [43]; A) [64]; B) [67]; C) [66]; D) n/a; E) [43].

**Insecticide-treated mosquito net:** Regulatory approval) [49]; A) [68]; B) [50]; C) [69]; D) [70]; E) [51].

**Rapid diagnostic test**: Regulatory approval) [71]; A) [8,72]; B) n/a; C) [69]; D) [73]; E) [72].

**Artemisinin-based combination therapy:** Regulatory approval) [74]; A) [75]; B) [76,77]; C) [69]; D) [74]; E) [78].

#### Milestone A - Coordinating architecture

The establishment of an organization or international partnership focused on supporting development, establishment of technical consensus around, and/or use of the intervention was considered consistent with a coordinating architecture. Hep B, Hib, PC, RV, RDT, and ACT were supported by product-development partnerships. ITNs were supported by the Roll Back Malaria Partnership.

#### Milestone B - Intervention designed for developing country health systems

Each vaccine and two of the malaria interventions were improved over time to better align with the needs of DCs. The year a new version of each intervention that was intentionally designed to meet the needs of DCs was approved by regulators, or equivalent, was considered relevant for availability. HepB and Hib antigens were combined with the widely implemented DTP vaccines to create new “four in one” or “five-in-one” vaccines. New versions of pneumococcal conjugate vaccines included additional serotypes prevalent in the developing world and improved packaging. New rotavirus vaccines decreased the size of packaging, required two doses instead of the traditional three, and were more heat stable. ITNs were replaced by LLINs, and an ACT specifically formulated and packaged for use in infants was developed after the initial tablet formulation. No major improvements in RDTs were identified over the course of their deployment. VIMS provided information on product presentation and formulation of Hib in most countries, used in this analysis to consider if the speed of scaling-up changed when the presentation of the vaccine was better aligned with the needs of DCs.

#### Milestone C - Financing commitment

A major global financing commitment by an international organization was considered important for affordability to countries to begin implementation. All of the interventions have been supported by such financing. From 2000, GAVI supported 72 to 75 countries with less than $1000 gross national income (GNI)/capita to purchase and implement vaccines. From 2011, it increased the threshold to $1500 GNI/capita decreasing eligibility to 56 countries due to inflation. For malaria control, the GFATM is by far the most important donor, supporting low and middle income countries.

#### Milestones D and E - WHO recommendations

Recommendations by WHO to use each intervention were considered important for international-level support for adoption. WHO issued an initial, limited recommendation and then a more comprehensive recommendation some years later for five of the interventions. The timing of the initial recommendation was milestone D, while the comprehensive WHO recommendation was milestone E.

### Statistical analysis

The year of country implementation of each intervention was extracted from the selected global databases into Microsoft Excel, and back-validated against the original databases for accuracy. Cox proportional hazard models were used to compare the rates of beginning implementation of interventions between countries, as functions of the intervention and income group of the country. The analysis for each country started with the year when the intervention was approved by a stringent regulatory authority, or equivalent. The data were treated as right-censored where the country had not introduced the intervention during the periods described previously. In this analysis the adoption rate corresponds to the hazard in a conventional survival analysis. Plots of the cumulative baseline hazard over time were used to assess time trends in the underlying rate of beginning implementation, allowing for the covariates of income level and milestones described previously. These analyses were carried out using the PHREG procedure in SAS (SAS Institute Inc., Cary, NC, USA, version 9.2 for Windows).

## Results

### Years to beginning implementation

The implementation of all interventions is presented in Table 4 for low and lower-middle-income countries after 5, 10 and 15 years. A decade after each studied vaccine or malaria intervention was approved by regulators, 33% or fewer low and lower-middle-income countries, and in most cases less than 15%, had begun to implement it. The exception was ACTs which 70% of low and 56% of lower-middle-income countries had begun to implement after 10 years.

**Table 4:** Percentage of LICs and LMICs implementing interventions after 5, 10, and 15 years

	**5 years**		**10 years**		**15 years**	
	**LIC**	**LMIC**	**LIC**	**LMIC**	**LIC**	**LMIC**
**Hepatitis B**	0%	6%	3%	24%	10%	41%
***Haemophilus influenzae*type B**	0%	0%	3%	0%	15%	26%
**Rotavirus**	0%	15%	--	--	--	--
**Pneumococcal**	0%	0%	5%	13%	--	--
**Insecticide-treated mosquito nets**	3%	5%	30%	33%	95%	72%
**Rapid diagnostic test**	0%	3%	10%	15%	--	--
**Artemisinin-based combination therapy**	18%	15%	70%	56%	--	--
**Average Vaccines**	0%	5%	4%	12%	--	--
**Average Malaria**	7%	8%	37%	35%	--	--
**Average All**	3%	6%	20%	24%	--	--

**Legend**.

LIC = Low-income countries; LMIC = Lower-middle-income countries.

The mean of the percentage of low-income countries beginning implementation of each intervention after five years was 3% and 20% after 10 years. However, malaria interventions were implemented sooner than vaccines, a mean percentage of 37% of countries at 10 years compared with only 4% for vaccines. No low-income country implemented any of the new vaccines in the first five years.

**Figure 3:**
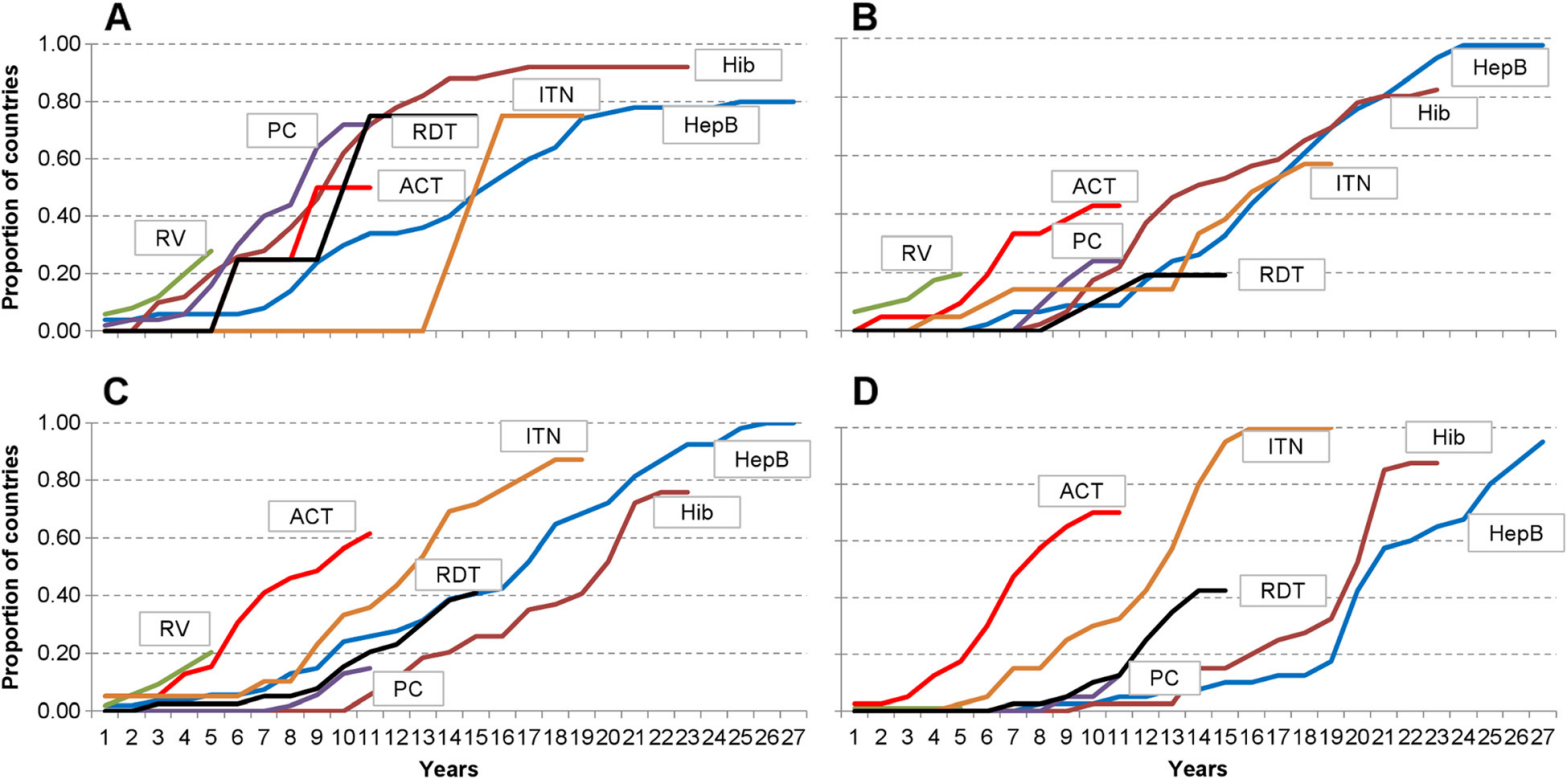
**Proportion of implementing countries over time in each income category, stratified by intervention.** The figure presents the proportion of countries implementing each intervention by year since regulatory approval. Panel **A** = High income countries; **B** = Upper middle income countries; **C** = Lower middle income countries; **D** = Low income countries. Color code: Hepatitis B vaccine (HepB) = Blue; *Haemophilus influenzae* type b vaccine (Hib) = Dark red; Rotavirus vaccine (RV) = Green; Pneumococcal vaccine (PC) = Purple; Artemisinin-based combination therapy (ACT) = Light red; Insecticide-treated mosquito net (ITN) = Orange; Rapid diagnostic test (RDT) = Black

The mean of the percentage of lower-middle-income countries beginning implementation of each intervention after five years was 6% and 24% after 10 years. However, malaria interventions were implemented sooner than vaccines, a mean percentage of 35% of countries at 10 years compared with only 12% for vaccines. Figure 3 shows the cumulative percentages of countries beginning implementation of each intervention, by number of years after regulatory approval and for each income group.

HepB, Hib and ITNs are approaching universal implementation, having been implemented by more than 80% of countries (Table 2). The mean time was 12.2 years across all malaria-endemic countries to begin implementing ITNs, and 14.6 years for Hib and 16.7 years for HepB across all countries (Table 5). Beginning implementation in low-income countries took an average of 12.2 years for ITNs, 18.8 years for Hib vaccine and 21.2 years for HepB vaccine as compared to 15.0, 9.0 and 13.2 years in high-income countries.

**Table 5:** Mean number of years (range) until countries began implementation, by income group

	**High-income**	**Upper- middle- income**	**Lower- middle- income**	**Low- income**	**All countries**
**Hepatitis B**	13.3 (1–25)	16.9 (6–24)	16.0 (1–26)	21.2 (8–27)	16.7 (1–27)
***Haemophilus influenzae*type B**	9.0 (3–17)	14.3 (8–23)	17.5 (11–22)	18.8 (10–22)	14.6 (3–23)
**Insecticide-treated mosquito net**	15.0 (14–16)	12.9 (4–18)	11.7 (1–18)	12.2 (5–16)	12.2 (1–18)

**Legend**.

The table shows data only for the three interventions which more than 80% of all countries (HepB, Hib), or malaria-endemic countries (ITNs), have begun to implement.

**Figure 4:**
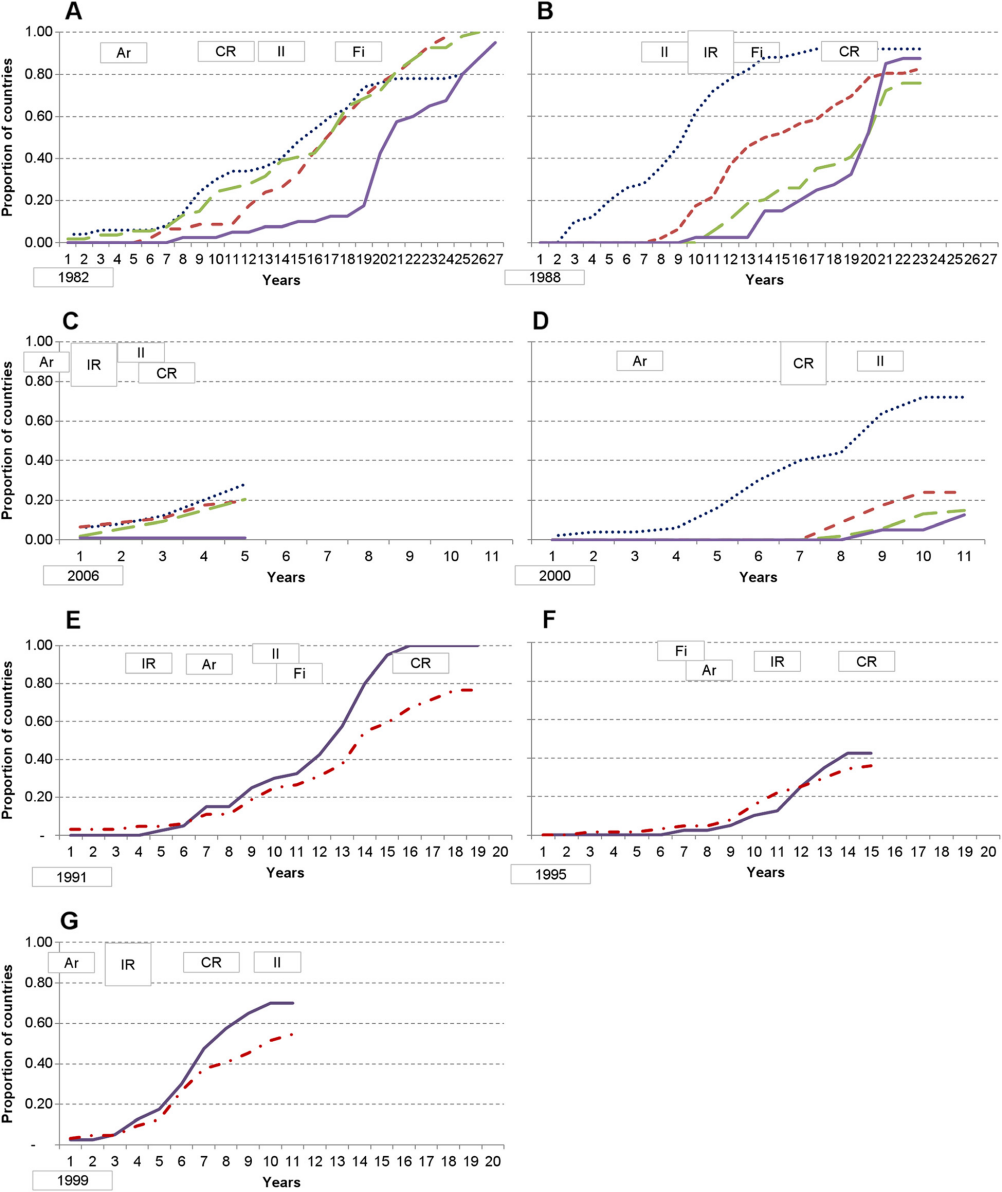
**Beginning implementation of each intervention by countries, by income group, including hypothesized milestones.** Panels **A**-**G** present the proportion of countries beginning to implement each intervention by year since the year of regulatory approval. Panel **A** = Hepatitis B vaccine; **B** = *Haemophilus influenzae* type b vaccine; **C** = Rotavirus vaccine; **D** = Pneumococcal conjugate vaccine; **E** = Insecticide-treated mosquito net; **F** = Rapid diagnostic test; and **G** = Artemisinin-based combination therapy. For vaccines, countries are stratified according to World Bank income groups: High = Blue dotted line; Upper‐ middle = Red short dashed line; Lower‐ middle = Green long dashed line; Lower = Purple line. Malaria-endemic countries are stratified by low income versus all other endemic countries. LICs = Purple line; Other endemic countries = Red dash and dot line. Year of regulatory approval (year 0) is provided in the bottom left hand corner of each panel. *Ar* indicates establishment of a group providing coordination (i.e. architecture). *II* indicates availability of an improved intervention better aligned with the needs of developing countries. *Fi* indicates year of a global financing commitment, such as through GAVI or GFATM. *IR* indicates year of initial WHO recommendation. C*R* indicates year of comprehensive (e.g. global) WHO recommendation

Figure 4 presents the cumulative implementation of each intervention by countries, stratified by income group. Twenty-seven years after HepB vaccine was first approved, 93% of countries had begun to implement it for routine infant use, with low income countries appearing to accelerate implementation after GAVI began to provide financial support. A few HICs in Europe recommend it instead for adolescents or high-risk individuals. It has taken more than 20 years for 90% of low-income countries to use Hib, with implementation in LICs appearing to accelerate after a comprehensive WHO recommendation. The lowest coverage of Hib (76% of countries) was in the lower-middle-income countries, many of which are too wealthy to receive financing from GAVI.

For rotavirus vaccines, 15-20% of high, upper-middle, and lower-middle-income countries had begun to implement it after five years, an equality across country income levels that did not occur for other vaccines. It took more than 10 years for Hib and pneumococcal vaccines to reach a similar proportion of lower-middle-income countries. Upper-middle-income countries appear to have implemented Hib and pneumococcal vaccines in the same proportions, with the first countries beginning implementation after seven years and reaching approximately 20% of countries at ten years. No low-income country had implemented rotavirus vaccine as of March 2011, more than five year after regulatory approval, which appears consistent with HepB, Hib, and PC vaccines where low-income early adopters did not begin implementation until 8–10 years after the vaccines were available.

Malaria interventions were initially implemented by similar proportions or a slightly higher proportion of wealthier countries. However, six years after approval, ITNs and ACTs began to be used in notably greater proportions of low-income than wealthier countries. After 12 years, a greater proportion of low-income countries had begun implementing RDTs than wealthier countries. These findings are consistent with the differences in disease burden in these countries, with higher levels in the poorest countries.

There were significant statistical differences in rates of beginning implementation between interventions relative to hepatitis B vaccine in high income countries (likelihood ratio statistic (LRS): 30.6; 6 degrees of freedom (d.f.); P < 0.001, adjusted for effects of income level and of the different milestones) (Table 6). ACTs was the fastest intervention to begin implementation, with a rate 2.41 times that of HepB (95% CI 1.38-4.21) (Table 6). Rotavirus is being implemented at 1.99 times that of HepB, but the confidence interval is wide and includes zero (95% CI 0.85-4.66). The slowest was RDTs with a rate of 0.54 (95% CI 0.33-0.88). Hib, PC, and ITNs all had similar rates to HepB.

**Table 6:** Relative rates of beginning implementation by intervention and country income (Cox proportional hazard)

	**Rate of beginning implementation**	**95% confidence interval**
***Haemophilus influenzae*type B vaccine**	0.81	(0.60-1.08)
**Rotavirus vaccine**	1.99	(0.85-4.66)
**Pneumococcal vaccine**	1.18	(0.70-1.98)
**Insecticide-treated mosquito nets**	0.96	(0.68-1.34)
**Rapid diagnostic test**	0.54	(0.33-0.88)
**Artemisinin-based combination therapy**	2.41	(1.38-4.21)
**Low-income**	0.51	(0.40-0.64)
**Lower-middle-income**	0.56	(0.44-0.70)
**Upper-middle-income**	0.52	(0.41-0.67)

**Legend**.

Rates of beginning implementation are calculated relative to the rate of beginning HepB implementation in high income countries in the absence of any of the facilitating milestones. All likelihood ratio statistics (interventions having 6 degrees of freedom and income groups having 3) testing these effects were highly significant, with P < 0.001.

There was a highly significant difference between level of income of countries and the rate of beginning implementation (LRS 27.2; 3 d.f.; P < 0.0001), this difference being almost entirely accounted for by the difference between high income countries and the others. There was very little difference in rates between the different categories of middle income countries, or between middle and low income countries (Table 6); each of these categories was associated with rates of beginning implementation only just over half that of high income countries.

### Milestones

Table 3 summarizes milestones for each intervention. Figure 4 indicates when each milestone from Table 3 was reached relative to regulatory approval (i.e. time zero). Patterns of temporal associations between access milestones, and between milestones and beginning implementation, can be drawn from Figure 4.

#### Milestone A - Coordinating architecture

A coordinating architecture was put in place years prior to significant beginning of implementation, except for Hib and ITNs. Coordination was in place years before comprehensive WHO recommendations were made in all situations, and prior to initial WHO recommendations in all cases except for that of ITNs. It was also in place before a financing commitment in all cases, except RDTs and before an improved intervention in all cases except Hib.

#### Milestone B - Intervention designed for developing country health systems

Less than 10% of low-income countries began implementation of any of the vaccines prior to an improved intervention being available. No lower or upper-middle-income countries began implementing Hib prior to availability of an improved vaccine, but up to 40% of countries were already implementing HepB and 10-20% were implementing rotavirus and/or pneumococcal vaccines. For HepB and Hib vaccines it was possible to estimate the average time from beginning implementation until coverage matched DTP3 levels (data not shown). After combination vaccines became available in 1997, which allowed the antigens to be administered in a single injection with DTP, countries required approximately one year less for coverage to be scaled-up to match DTP than it did prior to a combined vaccine. Improved malaria interventions became available after 30-70% of countries had already begun implementing each type of intervention. These data suggest that many LMICs, UMICs and HICs did not wait for an improved, second-generation vaccine prior to starting implementation. And that once the second generations were available, they may have been scaled-up more rapidly. Countries frequently did not wait for improved malaria interventions before beginning implementation.

#### Milestone C - Financing commitment

There was a sharp increase in the proportion of low-income countries beginning implementation of HepB and Hib vaccines and low and lower-middle-income countries implementing pneumococcal vaccines after GAVI’s advent and associated financing commitments. There was a similar increase in the proportion of countries, particularly low-income, beginning implementation of malaria interventions after GFATM’s advent and associated financing commitments. For ITNs and Hib, financing commitments came after an initial WHO recommendation, but before a comprehensive recommendation. RDT financing came before any recommendation, and for RV, PC and ACTs, a recommendation and financing came in the same year. A financing commitment generally preceded a sharp increase in the proportion of countries beginning implementation.

#### Milestones D and E - WHO recommendations

The proportion of countries beginning implementation of HepB, Hib, PC, ITNs, and ACTs did not accelerate in low, lower-middle and upper-middle-income countries until after a WHO recommendation. Comprehensive WHO recommendations for use of the malaria interventions did not come until 40% or more of low-income countries had begun to implement the interventions, while for HepB, RV and PC, comprehensive recommendations came prior to any significant implementation in LICs.

**Figure 5:**
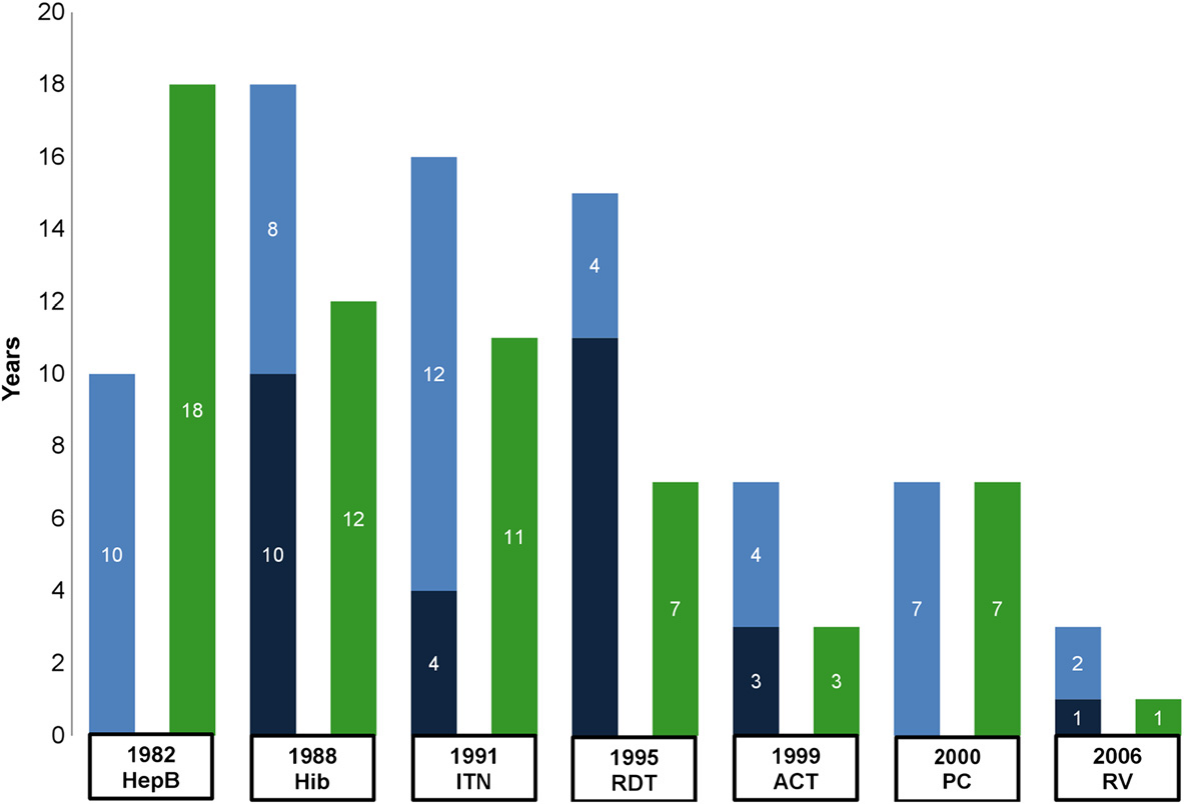
**Time from regulatory approval to WHO recommendation and financing, by intervention.** Interventions are presented from earliest to most recent year of regulatory approval. The year of regulatory approval and intervention name are indicated below each set of bars. Dark blue bars indicate the number of years to an initial recommendation, when relevant, while light blue bars indicate the number of years to a comprehensive recommendation. Green bars indicate the number of years to a financing commitment

Figure 5 shows the number of years from regulatory approval to initial and/or comprehensive WHO recommendation, and financing commitment, with the interventions listed in order from earliest to most recent year of regulatory approval. Five interventions had initial recommendations, which took a mean of 5.8 years (range 1–11). The additional time for those five interventions to receive a comprehensive recommendation was a mean of 6.0 years (range 2–12). HepB and PC had no initial recommendations, requiring 10 and 7 years respectively for WHO to issue comprehensive recommendations. For all seven interventions, the average time from regulatory approval to comprehensive recommendation was 10.9 years, with a range of 3–18 years. On average it took 8.4 years, with a range of 1–18 years, from regulatory approval to a financing commitment. The time required from regulatory approval to policy recommendations and financing commitments has decreased over the 22 year period represented by the interventions.

An initial proportional hazard model analyzed the relative rates associated with each of the milestones separately, adjusting for levels of income groups of countries and interventions. Initial WHO recommendation (relative uptake rate 1.83; 95% confidence interval (CI) 1.26-2.64), coordinating group (relative uptake rate 2.19; CI 1.45-3.30), and financing commitment (relative uptake rate 1.78; CI 1.30-2.45) were all positively associated in this analysis, suggesting these where associated with countries beginning implementation earlier than occurred for HepB. On the other hand, a comprehensive WHO recommendation (relative uptake rate 0.45; CI 0.33-0.61) was negatively associated, suggesting it was associated with countries taking longer to begin implementation than with HepB. Availability of an improved intervention seemed to have little effect on the rate of beginning implementation (relative uptake rate 0.86; CI 0.62-1.18). Since the timing of these milestones was unlikely to be independent, a further analysis was conducted in which the effects were fitted simultaneously to adjust for possible confounding (Table 7). In this analysis the estimated effect sizes were similar to those in the unadjusted analysis, while the only statistically significant milestones were the positive effect of initial WHO recommendation (1.97. 95% CI 1.33-2.94), and the association of a comprehensive WHO recommendation with a slowing down of beginning implementation relative to HepB (0.45; 95% CI 0.31-0.64).

**Table 7:** Effects of hypothesized access milestones on rate of beginning implementation (Cox proportional hazard)

	**Relative rate of beginning implementation**	**95% confidence interval**	**Likelihood ratio statistic**	**P-value**
**Coordinating group**	1.20	(0.74-1.95)	0.6	0.5
**Improved intervention**	0.85	(0.59-1.22)	0.8	0.4
**Financing commitment**	1.24	(0.88-1.75)	1.5	0.2
**Initial WHO recommendation**	1.97	(1.33-2.94)	11.2	<0.001
**Comprehensive WHO recommendation**	0.45	(0.31-0.64)	19.1	<0.001

**Legend**.

Adoption rates are calculated relative to the rate of adoption of HepB vaccine in high income countries.

**Figure 6:**
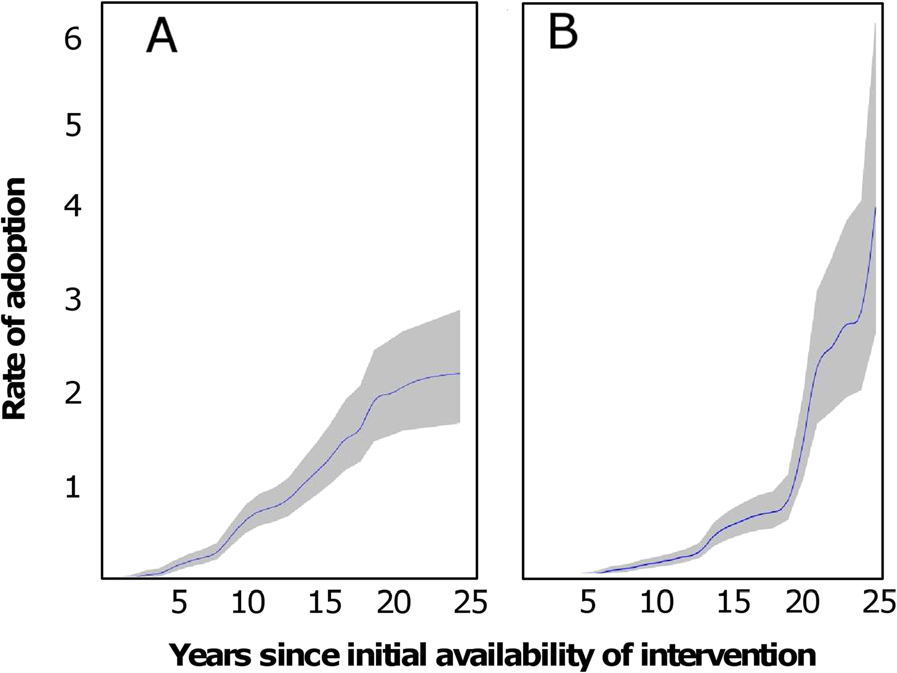
**Effect of time since regulatory approval on rate of beginning implementation.** The vertical axis shows the rate of beginning implementation (i.e. adoption) of interventions according to the number of years since regulatory approval. All interventions are included, except where too few countries were relevant to the analysis, as noted below. The grey area indicates the 95% confidence region around the result. A. High income countries only. Analysis includes all interventions except those against malaria. B. Low income countries only. Analysis includes all interventions except rotavirus vaccination

Plots of the rate of beginning implementation (i.e. rate of adoption; cumulative baseline hazard from the Cox model) for analysis including only high income countries (Figure 6A), suggested a more or less linear increase with time. This corresponds to a constant underlying rate of beginning implementation, once the effects of the different milestones are allowed for. In contrast, the cumulative baseline hazard for low income countries (Figure 6B) increased with the time for which the intervention had been available, indicating a tendency for interventions to be more likely to be implemented the longer they were available, even allowing for the effects of the milestones.

## Discussion

This paper analyzed the time between regulatory approval and beginning implementation of seven interventions; four vaccines and three malaria interventions. The interventions target diseases responsible for a substantial proportion of the overall global burden of disease, particularly for children in developing countries. According to WHO, each of these interventions should target a similar group of children, and be used globally in all countries or all malaria-endemic countries. We hypothesized that five milestones, consistent with the literature on access to interventions, were associated with the beginning of implementation in developing countries.

We found that during the five years after regulatory approval, interventions began to be used in only a tiny minority of developing countries. After 10 years, less than 1/3 of low and lower-middle-income countries had begun to use interventions. ACTs were the exception, being implemented earlier than the other interventions, likely due to the evolving crisis as resistance grew to antimalarial treatments. Even so, one may question if implementation of ACTs was fast enough. Vaccines and ITNs continue to be implemented in developing countries at approximately the same pace as HepB in the 1980s and 1990s, while RDTs are being implemented slower than HepB.

There is no international agreement on how quickly new interventions should be implemented. One option is to suggest that they should be introduced with minimal delay relative to the pace in high income countries. But that overlooks the much greater burdens of disease and numbers of lives lost in low income countries. These interventions should be implemented as fast as or faster than HICs. It is also hopeful that there appear to be shorter delays between regulatory approval and policy and financing decisions for more recent interventions. However, the history for RV, the most recent intervention, is more tangled than the more linear processes of the other interventions. During 2011, there was substantial, growing momentum through GAVI for use of rotavirus and pneumococcal conjugate vaccines [79]. This momentum cannot save the lives already lost over the many years that these vaccines have been available and not implemented, but hopefully the proportion of implementing countries will accelerate as suggested in Figure 6B.

The initial WHO recommendation and a financing commitment were most temporally associated with increasing the percentage of countries beginning implementation. A coordinating architecture was in place years before an increase in the percentage of countries beginning implementation for five of the interventions, suggesting it may contribute to but not trigger implementation. It appears that countries frequently did not wait for an improved intervention or a comprehensive WHO recommendation. This suggests that activities strengthening country decision-making may accelerate implementation.

Statistically, only the initial WHO recommendation was associated with beginning implementation in all analyses. This may not be surprising as countries and financing bodies often follow WHO’s guidance. This suggests that those developing new interventions should work systematically to anticipate the research questions WHO and international organizations, including financing bodies, will need addressed to make policy decisions. A more comprehensive WHO recommendation was associated with slower implementation. Wealth impacted implementation, differentiating high-income countries from all the developing countries which began implementation at slower, more similar rates.

### Challenges and limitations to the analysis

The analysis focuses on implementation of interventions through national health systems as a way of reaching enough people to achieve an equitable public health impact, consistent with the definition of access. Future analyses could consider ways to reflect private sector provision, which is relatively modest for vaccines but can be significant for malaria interventions. Analyses could also consider ways that political pressure and advocacy inform and influence decisions (this may have especially speeded up the introduction of ACTs), such as through key opinion leaders or the media.

The analysis did not consider country characteristics apart from income group, given that each of the interventions has been recommended for use globally. The analysis is also likely to be conservative, understating the delays, given that it focused on beginning of implementation, not the time to reach nation-wide coverage. Coverage is already analyzed by WHO and others elsewhere [57,80].

Data used in the analysis are imperfect. However, we found no evidence of a systematic reporting gap or bias from any group of countries. Imperfect data were partially offset by including every country in the world. The only relatively small income group was HIC malaria-endemic countries, of which there are only four (Table 2). It is important to recognize that countries can choose not to implement any intervention, or use one in a different fashion which may not be captured in databases. Data on the year of beginning implementation of interventions ideally would be taken from multiple sources (e.g. surveys, WHO, national data.) Multiple sources would be particularly important if the analysis focused on coverage rates, for which precise estimates are difficult to confirm [28]. Using few data sources was mitigated by limiting the analysis to the year of beginning implementation, a single time point which should be more reliably reported. Relying primarily on WHO data also has the benefit of limiting variability associated with incorporating data from differently designed studies.

We combed a broad selection of literature to establish the timing of milestones for each intervention. The milestones for rotavirus vaccine and ITNs were perhaps the most challenging. As noted in the methods, a RV vaccine was first approved in 1998 and then withdrawn by the manufacturer due to safety concerns. A new rotavirus vaccine was licensed by Mexico in 2004, an NRA that was not considered by WHO to be fully functional, and began to be implemented in Latin America. It was licensed by the EMA in 2006. It may be the case that the availability of a licensed vaccine from 1998 to 1999, and a new vaccine licensed by some countries from 2004 accelerated milestones and implementation planning. However it is also possible that the safety questions and shadow over RV vaccines slowed milestones and implementation. ITNs were deemed safe for use in 1991, which was considered the point of regulatory approval for this analysis. However, the first recommendation for use of ITN’s based upon demonstrated efficacy was made by WHO in 1995 for Africa [70]. International organizations did not develop a shared commitment to initiate large scale implementation of ITNs until 1997 and later. Bias due to investigator judgment was minimized by scrupulous attention to documentation of sources (Table 3).

There is no reason to assume that the pattern of delays in the adoption of vaccines and malaria interventions is unique. It is possible that the delays for other interventions are longer than those for immunization and malaria, which enjoy a relatively high profile within public health arenas.

### A revised hypothesis for accelerating access

One interpretation of the study’s findings is that the null hypothesis was disproved only for the initial WHO recommendation. However, the temporal associations and existing literature suggest caution in asserting that all the other milestones were not associated with developing countries beginning to implement interventions. An alternative interpretation, preferred by the authors, is that these milestones could be associated with countries beginning implementation if they took place earlier in the development and implementation of an intervention. And that by taking place earlier, they could shorten and increase the efficiency of the transition from R&D to decision-making and implementation (Figure 1).

This analysis leads to a revised hypothesis which could be evaluated as new interventions are being developed. The hypothesis suggests that organizations need to begin working systematically on access-related issues earlier on, during the R&D stages, in order to shorten the time to begin implementation and realize equitable access in developing countries. Carefully paced activities during R&D could lead to decision-making processes at international and country levels and milestones being achieved shortly after regulatory approval, as has been proposed recently for malaria vaccines [81,82].

**Figure 7:**
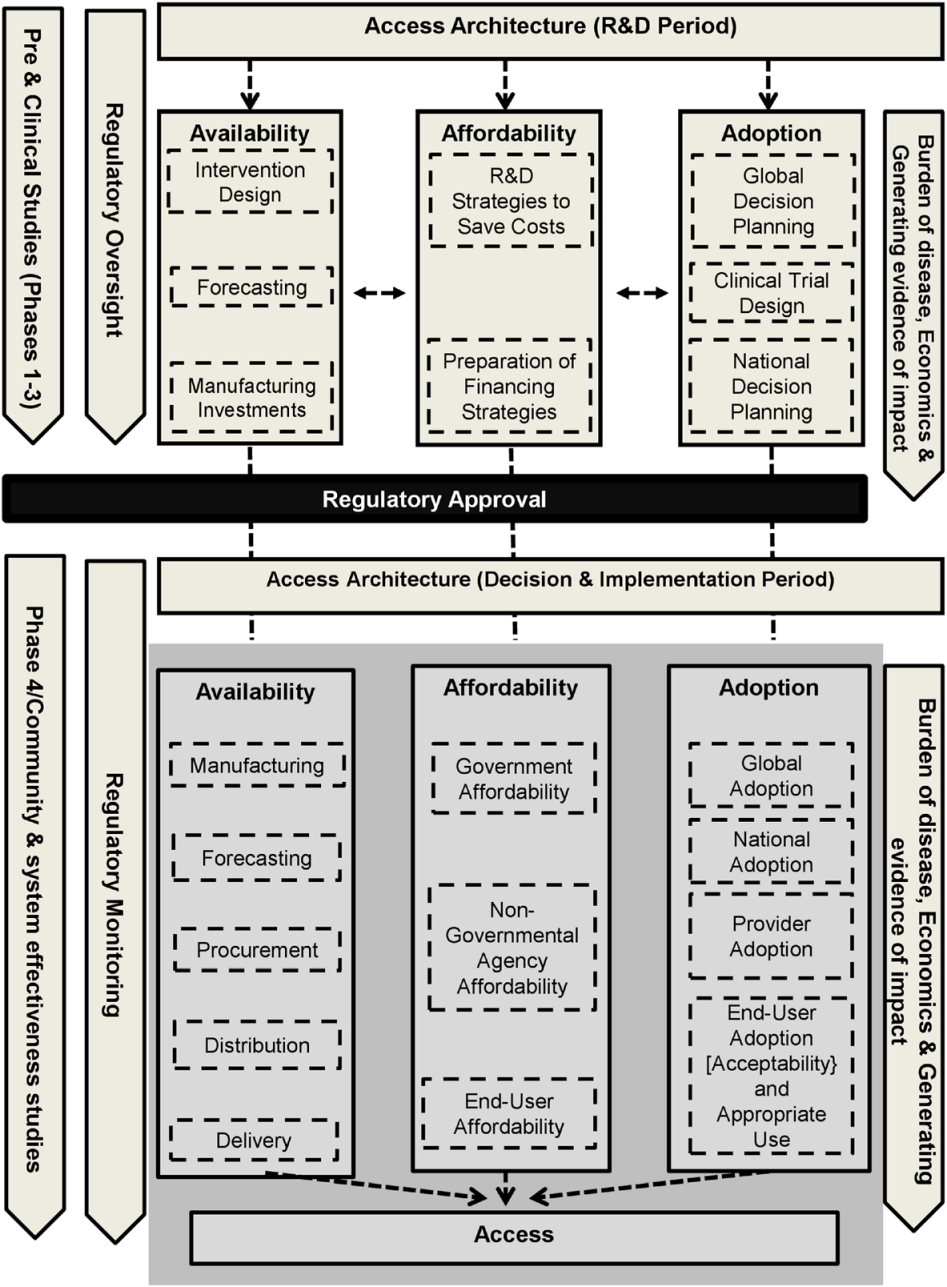
**Proposed access framework incorporating R&D and implementation periods.** The area in grey represents the original access framework as shown in Figure 2. Other areas are new to the framework. Actions that take place during the R&D period are described in the space above the black strip, “Regulatory Approval”, while actions carried out in the decision and implementation period are described in the space below. Area in grey is reproduced under a Creative Commons Attribution-Noncommercial-Share Alike 3.0 License [8]

Figure 7 builds on Frost and Reich’s (2008) access framework. It proposes additional activities specifically intended to take place prior to regulatory approval and thereby accelerate the beginning of implementation and eventual access. It must be noted that there are risks to planning for access during the R&D phase. Interventions can always fail due to safety, efficacy or other considerations.

The R&D process requires a coordinated, long-term and systematic approach to associated access activities; an approach which is specific to each intervention’s context. Available interventions need to be tailored to fragile developing country health systems and their users, and scientific questions resolved to facilitate eventual implementation. Affordability-related decisions during R&D seek to minimize future purchase and implementation costs, and incorporate interactions with international financing bodies, such as GAVI and the GFATM, years before funds are required. Adoption activities relate to planning by international organizations to identify and ensure that information needed for normative guidance and policy recommendations are developed at pace with, or as part of, clinical development [43].

A number of other activities that directly inform, and that may be directly informed by, access activities are reflected in the proposed framework. Regulatory oversight of clinical trials extends throughout R&D and evolves into regulatory monitoring and pharmacovigilance in the implementation period. Pre-clinical and clinical studies lead into phase four effectiveness studies and operational research. Epidemiological, economic and modeling studies may relate broadly to a disease area and/or may directly inform decisions on use of a specific intervention.

## Conclusion

The analysis in this paper suggests that 10 years or more after a proven life-saving public health intervention becomes available, only a small fraction of countries in need in the developing world is likely to have access to it. As a result, lives are being lost unnecessarily. Investments in carrying out high-quality clinical trials and regulatory processes as fast as possible are at risk of being wasted. During this extended period, manufacturers may be left with idle capacity or may decide to exclude low and lower-middle-income countries entirely from their supply plans, in favor of more reliable markets in high-income countries.

This paper hypothesizes that the international community can accelerate implementation and access by systematically addressing access-related topics during the R&D period of new interventions. Such an approach raises challenges for organizations, asking them to identify ways they can contribute to new interventions during R&D, anticipating and beginning to address likely bottlenecks in advance. Identifying the key areas for early foresight in the development process has the potential to significantly shorten the time elapsed before developing countries benefit from new interventions, and as a consequence, reduce the unnecessary disease and loss of life that is experienced today.

## Abbreviations

ACT: Artemisinin-based combination therapies for treating malaria; *Ar*: Year of establishment of a group providing coordination (i.e. architecture); DC: Developing country; EMA: European Medicines Agency; *Fi*: Year of a global financing commitment, such as through GAVI or GFATM; GAVI: GAVI Alliance; GFATM: Global Fund to Fight AIDS, Tuberculosis and Malaria; *CR*: Year of comprehensive (i.e. global) WHO recommendation; HepB: Hepatitis B vaccine; Hib: *Haemophilus influenza*e type b vaccine; HIC: High-income country (World Bank defined); IPTi: Intermittent preventive treatment for malaria in infants; *II*: Year of availability of an improved intervention better aligned with the needs of developing countries; *IR*: Year of initial WHO recommendation; ITN: Insecticide treated net; LIC: Low-income country (World Bank defined); LLIN: Long-lasting insecticidal nets; LMIC: Lower-middle-income country (World Bank defined); PC: Pneumococcal conjugate vaccine; PDP: Product development partnership; RV: Rotavirus vaccine; R&D: Research and development; RDT: Rapid diagnostic test; UMIC: Upper-middle-income country (World Bank defined); WHO: World Health Organization.

## Competing interests

AB, TS, DdS, and CL have no competing interests.

## Authors’ contributions

AB developed the analysis strategy with input from TS, DdS, and CL. AB conducted the analysis and drafted the manuscript. TS conducted the statistical analysis. TS, DdS, and CL reviewed and critically edited the manuscript. All authors read and approved the final manuscript.

## Pre-publication history

The pre-publication history for this paper can be accessed here:

http://www.biomedcentral.com/1471-2458/12/683/prepub
